# A manually curated annotation characterises genomic features of *P. falciparum* lncRNAs

**DOI:** 10.1186/s12864-022-09017-2

**Published:** 2022-11-30

**Authors:** Johanna Hoshizaki, Sophie H. Adjalley, Vandana Thathy, Kim Judge, Matthew Berriman, Adam J. Reid, Marcus C. S. Lee

**Affiliations:** 1 Wellcome Sanger Institute, Wellcome Genome Campus, Hinxton, Cambridge, CB10 1SA UK; 2 Micrographia Bio, London, W12 0BZ UK; 3 MRC Weatherall Institute of Molecular Medicine, University of Oxford, Oxford, OX3 9DS UK; 4 Present address: Department of Microbiology and Immunology, Columbia University Medical Center, New York, NY10032 USA; 5 Wellcome Centre for Integrative Parasitology, University of Glasgow, Glasgow, G12 8TA UK; 6 Present address: Wellcome/Cancer Research UK Gurdon Institute, University of Cambridge, Cambridge, CB2 1QN UK

**Keywords:** lncRNA, Noncoding, Annotation, Manual curation, *Plasmodium falciparum*, long-read RNA sequencing.

## Abstract

**Background:**

Important regulation occurs at the level of transcription in *Plasmodium falciparum* and growing evidence suggests that these apicomplexan parasites have complex regulatory networks. Recent studies implicate long noncoding RNAs (lncRNAs) as transcriptional regulators in *P. falciparum*. However, due to limited research and the lack of necessary experimental tools, our understanding of their role in the malaria-causing parasite remains largely unelucidated. In this work, we address one of these limitations, the lack of an updated and improved lncRNA annotation in *P. falciparum*.

**Results:**

We generated long-read RNA sequencing data and integrated information extracted and curated from multiple sources to manually annotate lncRNAs. We identified 1119 novel lncRNAs and validated and refined 1250 existing annotations. Utilising the collated datasets, we generated evidence-based ranking scores for each annotation and characterised the distinct genomic contexts and features of *P. falciparum* lncRNAs. Certain features indicated subsets with potential biological significance such as 25 lncRNAs containing multiple introns, 335 lncRNAs lacking mutations in *piggyBac* mutagenic studies and lncRNAs associated with specific biologic processes including two new types of lncRNAs found proximal to *var* genes.

**Conclusions:**

The insights and the annotation presented in this study will serve as valuable tools for researchers seeking to understand the role of lncRNAs in parasite biology through both bioinformatics and experimental approaches.

**Supplementary Information:**

The online version contains supplementary material available at 10.1186/s12864-022-09017-2.

## Background

The advent of genome sequencing has dramatically impacted research on malaria, a disease that has afflicted humans for millennia and continues to cause 241 million infections and 627, 000 deaths annually [1]. Malaria is caused by infection with the *Plasmodium* protozoan parasite, which is transmitted to humans through bites from infected mosquitoes. Of the species that cause human disease, *Plasmodium falciparum* is the most common cause of life-threatening malaria [1]. The study of the parasite’s biology relies heavily on the genome assembly and its annotation [2]. It has improved our understanding of gene expression and regulation and led to new insights into virulence, evolution, population diversity and drug resistance [3–5]. However, while genomic features such as protein-coding genes are relatively well-annotated, the role of non-coding transcription in the parasite’s biology remains poorly understood. In particular, one class, the long noncoding RNAs (lncRNAs), has yet to be fully described [6].

Defined as being at least 200 base pairs (bp) long, most lncRNAs undergo post-transcriptional processing (capping, splicing and polyadenylation) and form secondary and tertiary structures that can bind DNA, RNA and proteins [7]. Through these interactions, lncRNAs can act as transcriptional regulators. They regulate gene expression via various mechanisms such as acting as DNA enhancers or scaffolds for transcription initiation machinery, binding transcription factors, sequestering miRNAs, recruiting chromatin modifiers, interfering with mRNA splicing or stability, modulating signalling pathways or nuclear organisation [7, 8]. Whereas these interactions often occur in nearby genes located upstream or downstream of the lncRNA (*cis-*regulation), lncRNAs can also regulate distant genes (*trans-*regulation) through diffusion or chromatin conformations. Extensive research elucidating the roles of mammalian lncRNAs such as Xist, HOTAIR, FIRRE, lncRNA p21, Malat1, NEAT1, etc. and their implications in disease has revealed the vast range of mechanisms by which lncRNAs regulate gene expression in diverse biological contexts and inspired new approaches for therapeutics [9–11].

LncRNAs were first identified in *P. falciparum* when they were associated with members of the *var* multi-gene family, which encode PfEMP1, a variant antigen expressed on infected erythrocytes [12]. Early lncRNA annotations by Broadbent et al. and Liao et al. identified 60 lncRNAs through DNA tiling arrays and a further 147 lncRNAs from computational analysis of short-read RNAseq, respectively [13, 14]. Studies identifying pervasive antisense transcription in *P. falciparum* further supported the existence of lncRNAs such as the Siegel et al. study that identified 1247 genes with natural antisense transcription [15–20]. A later study by Broadbent et al. using strand-specific short-read RNAseq generated an annotation of 1134 lncRNAs in the parasite genome [21]. The lncRNA sequences differed from protein-coding sequences in having reduced G + C content, increased repetitive sequences, fewer introns, and lower transcript expression and stability [21]. The lncRNAs also exhibited stage-specific expression that correlated with the expression of neighbouring and overlapping genes, leading Broadbent et al. to propose a regulatory role for lncRNAs in *P. falciparum* transcription [21].

Further studies provided additional evidence of the regulatory role of lncRNAs and their implication in key biological processes [18, 22–25]. For instance, the expression of antisense lncRNAs in *var* introns has been associated with *cis* activation of *var* genes and consequently, *var* gene switching, a mechanism of immune evasion [26]. However, not all *var* genes are regulated in this way because the *var2csa* intron can be deleted and yet, still be activated and silenced [27]. Another example of transcriptional control by lncRNAs is found in the regulation of sexual differentiation. *gdv1* is an upstream activator of sexual commitment and when expressed, the GDV1 protein evicts the epigenetic silencer HP1 from its specific loci [22]. The expression of *gdv1* is negatively regulated by an antisense lncRNA during blood stages. When the antisense locus is disrupted, the expression of GDV1 is increased, leading to increased dissociation of HP1 from heterochromatin, consequently increasing the expression of *ap2-g,* a transcription factor that initiates sexual commitment [22]. LncRNAs associated with telomeres have been proposed to be regulators of telomere maintenance and chromatin remodelling. These lncRNA-TAREs (transcripts containing the telomere-associated repetitive elements) are enriched in the nuclear fraction. Among these, TARE6 has been shown to complex with histone H3 using a hairpin structure; however, it is not known whether this interaction affects gene regulation [25]. Although the aforementioned studies provide insights into how lncRNAs may regulate biological processes, many recent reviews highlight that these examples represent only a small subset of the thousands of lncRNAs identified so far in *P. falciparum* [6, 28–30].

One challenge that has stalled the large-scale characterisation of lncRNAs is the lack of an updated *P. falciparum* lncRNA annotation. Since the publications of *P. falciparum* lncRNA annotations, there have been significant updates in the annotations of UTRs in *P. falciparum* and advances in sequencing technologies that are more suitable for lncRNA detection. Previous transcriptional studies have predicted additional transcripts as potential lncRNAs however, these datasets have not been used to generate annotations i.e. with collapsed reads and consensus start and stop coordinates [18, 31, 32]. Annotation of lncRNAs is made difficult by the low read coverage of short sequencing reads that map to low-complexity regions and by the complexity of resolving overlapping expression from neighbouring transcriptional units. Long-read sequencing technologies from Pacific Biosciences (PacBio) and Oxford Nanopore Technologies (ONT) have extended RNA sequencing lengths and in the case of ONT enabled direct-RNA sequencing without the need for cDNA generation, amplification, or fragmentation. In *P. falciparum*, long-read RNAseq has proven effective for refining UTR annotations and analysing transcript isoforms in *P. falciparum* [31, 33]. In this work, we used long-read direct-RNA ONT sequencing and a collation of supportive datasets from the literature to manually generate a new lncRNA annotation for blood-staged *P. falciparum*. In addition to confirming 1250 lncRNAs, we identified and classified a further 1119 novel lncRNAs.

## Results

### The *P. falciparum* transcriptome contains over two thousand lncRNAs

To manually create a set of new lncRNA annotations, we generated new transcript sequencing data and compiled various existing datasets. We sequenced asexual intra-erythrocytic-staged *P. falciparum* 3D7 parasites using long-read RNA sequencing (Oxford Nanopore Technologies) (Table 1, Additional File 1: Supp. Table 1). Mixed stages were sequenced to capture the broad scope of lncRNA expression in the intra-erythrocytic cycle. LncRNAs tend to have low expression, therefore to improve read depth and gain additional confidence in detecting lncRNA transcripts, long-read RNA sequence data from the present study was collated with data from Lee et al. (Table 1, Additional File 1: Supp. Table 1) [33]. We also generated short-read RNA sequencing (Illumina) of synchronised asexual *P. falciparum* 3D7 parasites to support the annotations made from the long-read data (Additional File 1: Supp. Table 1). Furthermore, datasets from transcriptional start site (TSS) and chromatin accessibility (ATAC-seq) studies as well as existing lncRNA annotations were obtained from various sources (Table 1) [21, 31, 34–37].

**Table 1:** Datasets used for manual curation of *P. falciparum* lncRNA annotation

Use	Dataset	Type	Reference	Accession
Annotation	Pf nanopore 1	Nanopore long read	**This work**	E-MTAB-11766
	Pf nanopore 2	Nanopore long read	Lee et al. [33]	
Contextual support	Pf short read	Illumina short read	**This work**	ERP104547
	Pf transcription start site sequencing 1	Illumina short read	Chappell et al. [31]	
	Pf transcription start site sequencing 2	Illumina short read	Kensche et al. [34]	
	Pf transcription start site sequencing 3	Illumina short read	Adjalley et al. [35]	
	Pf ATAC-seq	Illumina short read	Ruiz et al. [36]	
	Pf ncRNA calls	Illumina short read	Chappell et al. [31]	
	Pf lncRNA annotation	Annotation	Broadbent et al. [21]	
	Pf ncRNA annotation	Annotation	PlasmoDB [37]	
Comparative analysis	Pf lncRNA annotation	Annotation	Liao et al. [14]	
	Pf genes with antisense transcripts	Gene list	Siegel et al. [18]	
	Pf ncRNA calls	Predicted transcripts	Chappell et al. [31]	
	Pf lncRNA calls	Predicted transcripts	Yang et al. [32]	

**Fig. 1:**
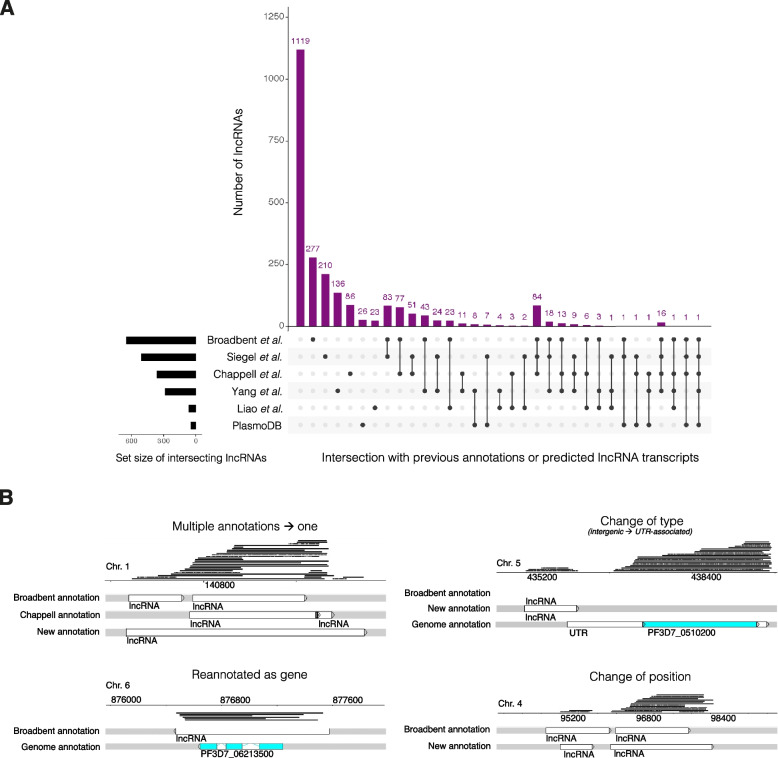
Verification of previous *P. falciparum* lncRNA annotations in the literature. Of the 2369 lncRNAs, 1119 were unique to this study and 1250 were previously annotated by Broadbent et al. or Liao et al., or predicted by Siegel et al.*,* Chappell et al., Yang et al.*,* and/or listed on PlasmoDB from various published studies [14, 18, 21, 31, 32, 37]. **a** An upset plot shows the number and membership of the previously annotated lncRNAs as well as the size of the set. Gene IDs from the Siegel et al. dataset were intersected with gene IDs of genes antisense to lncRNAs in this work [18]. **b** Snapshots demonstrate examples of changes to previous annotations

Annotation was completed by visualising and evaluating all the datasets in a genome browser and assigning an evidence-based ranking score for each annotation, with a 1 signifying the most supportive evidence and 9 the least (Additional File 2: Supp. Fig. 1, Additional File 3). We identified a total of 2369 lncRNAs in *P. falciparum* of which 1119 were novel to this study. The remaining 1250 were previously annotated by Broadbent et al. or Liao et al., listed on PlasmoDB (from various sources) or were predicted by Siegel et al., Chappell et al. or Yang et al. (Fig. 1A, Table 1) [14, 18, 21, 31, 32, 37]. Some previous annotations were updated; for example, long-read sequencing enabled the extension of lncRNAs that were previously partially annotated and the fusion of those previously annotated as multiple lncRNAs (Fig. 1B, Additional File 1: Supp. Table 2). LncRNA boundaries (start and stop positions) from previous annotations were updated in the new annotation to match the position of the outermost read in the collated long-read sequencing.

### LncRNAs are produced from distinct genomic contexts

**Fig. 2:**
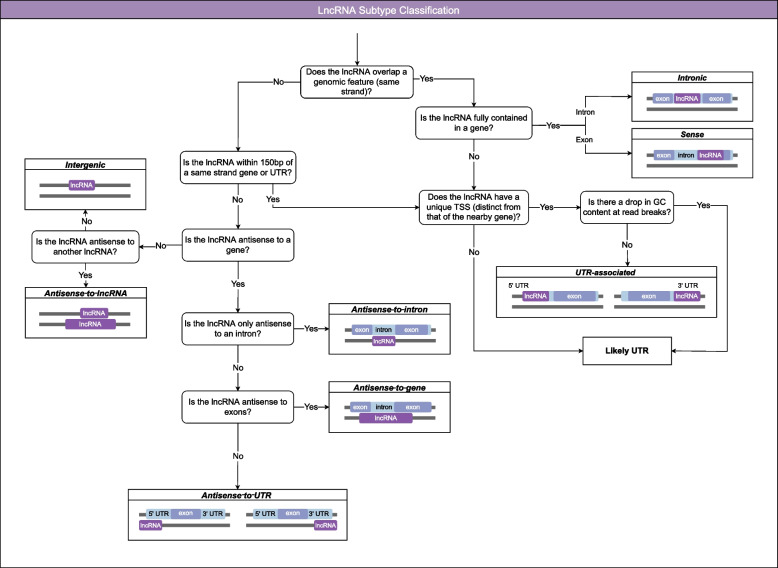
Schematic representation of the classification of lncRNA into genome context-based subtypes. Annotations were categorised by genomic context using a decision tree. LncRNAs that overlapped a gene on the same strand were classified as either *intronic* if contained within the intron or *sense* if contained within a single exon. No lncRNAs were annotated that spanned multiple exons in a gene. LncRNAs that overlapped a UTR and lncRNAs nearby genes (within 150 bp of an annotated UTR or exon or read from the gene) were flagged as potential *UTR-associated* lncRNAs. To delineate *UTR-associated* lncRNAs from UTR transcripts (that could be fragmented due to drops in GC content or alternative start sites) careful examination of collative data was performed. This included an analysis of the level of overlap between reads from the putative lncRNA and gene/UTR, the presence of a unique transcriptional start site (distinct from the gene) and the lack of evidence of a drop in GC content. LncRNAs that were antisense (opposite strand) to genomic features were classified based on the type of antisense genomic feature: *antisense-to-gene, antisense-to-intron, antisense-to-UTR* and *antisense-to-lncRNA*. The *antisense-to-intron* lncRNAs were contained within the intron boundaries (with little to no overlap with the exon). The *antisense-to-UTR* lncRNAs only overlapped the UTR, not the exons and the level of overlap varied. Some lncRNAs could be classified as multiple subtypes if overlapping multiple features – the classification has a hierarchy starting with: *intronic, sense, UTR-associated, antisense-to-intron, antisense-to-gene, antisense-to-UTR* and *antisense-to-lncRNA.* LncRNAs not overlapping, antisense to, or nearby (150 bp) any feature were classified as intergenic

**Fig. 3:**
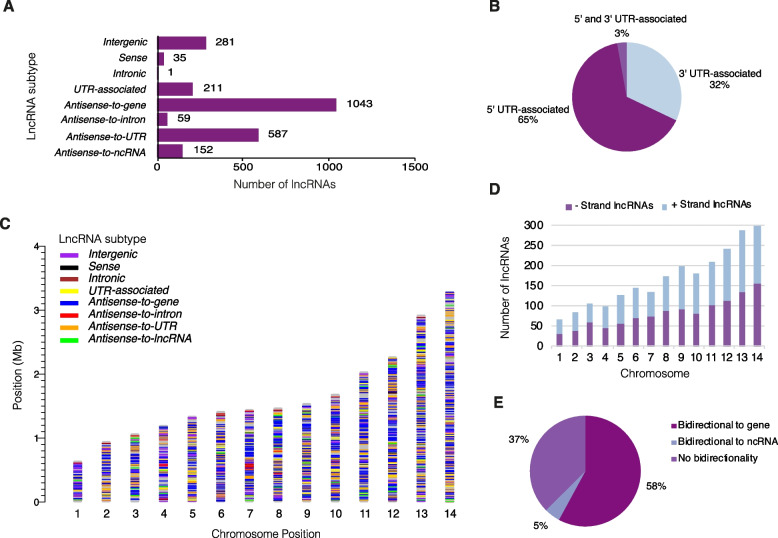
Genomic features of *P. falciparum* lncRNAs. **a** The majority of lncRNAs were *antisense-to-gene* lncRNAs or *antisense-to-UTR* lncRNAs, followed by *intergenic* lncRNAs, *UTR-associated* lncRNAs and *antisense-to-lncRNA* lncRNAs. A minority were *antisense-to-intron*, *intronic* or *sense* lncRNAs. **b** *Antisense-to-UTR* lncRNAs were most commonly (65%) associated with a 5′ UTR, followed by 32% associated with a 3′ UTR and a small subset (3%) were nestled between 5′ and 3′ UTRs. **c** LncRNAs and their genome-context subtypes were distributed throughout the genome. **d** LncRNAs were equally distributed between positive and negative strands and their abundance in chromosomes was relative to chromosome size. **e** For lncRNAs with an associated transcriptional start site, 65% had evidence of bidirectionality (transcription in both directions in the same location and time point)

LncRNAs were classified into eight subtypes based on genomic context: *intergenic*, *antisense* (to genes, UTRs, introns, or lncRNAs), *UTR-associated*, *intronic* and *sense* (within an exon) (Fig. 2, Additional File 3). The most common subtypes were *antisense-to-gene* (44%), followed by *antisense-to-UTR* (24.7%) and *intergenic* (11.9%) (Fig. 3A). The remaining subtypes were less common: *UTR-associated* (8.9%), *antisense-to-lncRNA* (6.4%), *antisense-to-intron* (2.5%), *sense* (1.5%) and *intronic* (0.04%). Of the *UTR-associated* lncRNAs, 65% were associated with a 5′ UTR, 32% were associated with a 3′ UTR and 3% were spanning two genes, associated with a 5′ and 3′ UTR (Fig. 3B).

The lncRNA subtypes were distributed throughout the chromosomes, but occasionally formed location-based clusters of 3–5 lncRNAs (Fig. 3C, Additional File 1: Supp. Table 3). There was no apparent strand preference for the production of lncRNAs with 49% on the negative strand and the remaining 51% on the positive strand (Fig. 3D). Previous research has suggested that the vast majority of promoters in *P. falciparum* are bidirectional, suggesting that the majority of lncRNAs may potentially be driven by gene promoters [35]. We determined if a bidirectional promoter was present by assessing if a TSS was on the opposite strand at the same location and same stage (time point) using the Chappell et al. dataset [31]. For the 2199 lncRNAs with evidence of an associated TSS, 70% had evidence of bidirectionality with 65% potentially sharing a promoter with genes, and 5% with other lncRNAs (Fig. 3E).

**Fig. 4:**
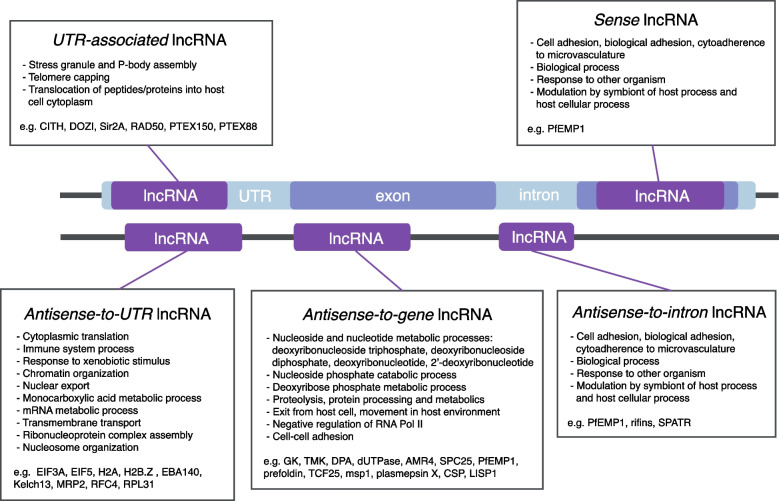
GO term enrichment of genes contextually-associated with *P. falciparum* lncRNA subtypes. Gene ontology (GO) term enrichment was completed on PlasmoDB using biological process ontology for genes associated with each lncRNA subtype: for s*ense* and *UTR-associated* lncRNAs, these were the overlapping genes (same strand) and for lncRNAs antisense to genomic features, these were the antisense genes [37]. Enrichment was determined based on the fold change and odds ratio calculated from the occurrence of GO terms in the set compared to the background. The *p*-value cut-off selected was 0.01. The GO terms were reduced using REVIGO [38]

We observed that the *sense* and *antisense-to-intron* lncRNAs were almost exclusive to *var* genes, where this configuration has been shown to be functionally relevant [23, 26]. We therefore completed a Gene Ontology (GO) term-enrichment analysis to investigate functional similarity between the genes contextually-associated with the lncRNAs (genes that overlapped for *sense* and *UTR-associated* lncRNAs, and antisense genes for *antisense-to-gene/UTR/intron* lncRNAs). For each subtype, significant enrichment (*P* < 0.01) of multiple GO terms was observed (Fig. 4). Matching our observations, genes associated with the terms *adhesion*, *response to other organisms*, and *modulation by symbiont of host process* (mainly *var* genes as well as other genes encoding surface-exposed proteins) were enriched in *sense* lncRNAs and *antisense-to-intron* lncRNAs. *Antisense-to-gene* lncRNAs (the largest classification) were enriched for genes involved in nucleoside and nucleotide metabolic and catabolic pathways along with protein metabolism, adhesion and movement in the host environment. *Antisense-to-UTR* lncRNAs were enriched for genes associated with chromatin organisation and translation machinery and *UTR-associated* lncRNAs were enriched for genes relating to stress granule and P-body assembly, telomere capping and translocation of proteins in the cytoplasm.

### Some lncRNAs contain structural RNA sequences

**Fig. 5:**
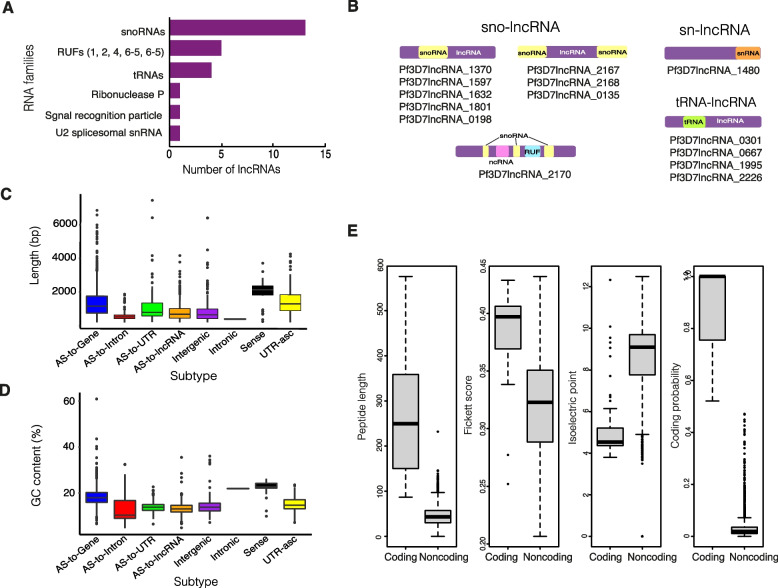
Sequence features of *P. falciparum* lncRNAs. **a** 26 RNA families (22 unique) were identified that aligned to annotated lncRNA sequences: 13 snoRNAs, 5 RUFs, 4 tRNAs, ribonuclease P, signal recognition peptide and U6 spliceosomal snRNA using Rfam [39]. **b** Visual representation and IDs of lncRNAs containing snoRNAs, snRNAs and tRNAs. **c** LncRNA lengths ranged from 200 to 7452 bp and the distribution of lengths differed between subtypes (AS:antisense to). **d** LncRNAs were AT-rich with an average GC content of 15.97% and the distribution of GC content differed between subtypes (AS:antisense to). **e** 16 lncRNAs were identified as putatively coding by sequence feature-dependent coding potential analysis with CPC2 [40]. The distributions of the measures used to determine coding potential (peptide length, isoelectric point and Fickett score) are presented for putative coding and noncoding lncRNA annotations

Searches against the RNA families database (Rfam) revealed that 19 lncRNAs contained sequences associated with 22 described RNA families (Fig. 5A, Additional File 1: Supp. Table 4), including those encoding known structural RNAs such as the signal recognition particle RNA, the ribozyme ribonuclease P and several RNAs of unknown function (RUFs) [41]. Additionally, some lncRNAs contained sequences corresponding to smaller RNAs (usually shorter than 200 nucleotides) including 13 snoRNAs, four tRNAs and one snRNA that we describe respectively as sno-lncRNAs, tRNA-lncRNAs and sn-lncRNAs (Fig. 5B, Additional File 3). LncRNAs containing structural RNA sequences have been previously identified in other organisms including humans (sno-lncRNAs) and plants (lncRNA containing a tRNA-like molecule) [42–44]. We also identified examples where more than one structural RNA sequence was contained within a single lncRNA. There were three examples where two snoRNAs flanked the ends of a single lncRNA, which resembles the structure of sno-lncRNAs in humans (Fig. 5B). There was also one example of multiple snoRNAs, a RUF and ncRNA forming a single RNA product (Pf3D7lncRNA_2170) (Fig. 5B). Cotranscription of snoRNAs at this locus has been previously suggested by Chakrabarti et al. [41].

### Several lncRNAs may code for small proteins

*P. falciparum* lncRNAs have an average length of 1146 bp (ranging from 200 bp to 7452 bp) and average GC content of 16%, lower than the GC content of the overall *P. falciparum* genome which is 19.4% but less than that of all non-coding regions, which approaches 10% [45]. However, both transcript lengths and GC content vary between subtypes (Fig. 5C, D). *Sense* lncRNAs, i.e. located within gene exons, displayed a clear bias towards both higher length distributions and greater average GC content. Similarly, *antisense-to-gene* lncRNAs had a higher average GC content compared to those lncRNAs found in non-coding regions of the genome. Like the Broadbent et al. study, we noted that it is uncommon for *P. falciparum* lncRNAs to contain introns, with only 5% detected in our analysis [21]. Of these lncRNAs, most were *antisense-to-gene* lncRNAs (59%) and the rest consisted of other subtypes. Broadbent et al. previously highlighted lncRNAs that contain multiple introns as notable due to the rareness of this property [21]. In addition to the three examples they highlighted (lncRNAs close to *gdv1*, *etramp9* and rRNA methyltransferase), we identified a further 25 lncRNAs that share these features (Additional File 3). Two examples, which are antisense to PF3D7_1115200 (SET7) and conserved protein PF3D7_0918400 (unknown function) are shown here (Additional File 2: Supp. Fig. 2).

Some apparent lncRNAs might in fact be protein-coding genes that have been missed in previous annotations. In particular, open reading frames (ORFs) encoding small proteins or peptides are hard to identify [46]. Therefore, we calculated the coding potential of each lncRNA using the coding potential calculator algorithm CPC2, which can be used for non-model organisms without the need to retrain the model [40]. CPC2 uses four sequence-intrinsic features to predict the coding probability of RNA transcripts: Fickett score, ORF length, ORF integrity and isoelectric point. As expected, the vast majority of lncRNAs were predicted to be noncoding transcripts. However, 16 lncRNAs were determined to have the potential to encode proteins and warrant further investigation (Fig. 5E, Additional File 1: Supp. Table 5). Most of these putative proteins were 100–150 amino acids in length and when queried in the Caro et al. *P. falciparum* ribosomal profiling dataset, most had some modest evidence of ribosomal footprints although often not spanning the length of the lncRNA and would require further experimental validation (Additional File 1: Supp. Table 5) [47]. Only two shared similarities with other proteins: Pf3D7lncRNA_1391, a lncRNA antisense to PF3D7_1116500 (folate transporter 2), and Pf3D7lncRNA_0624, an intergenic lncRNA, shared similarity with predicted proteins in other *P. falciparum* strains like Dd2 (Additional File 2: Supp. Fig. 3).

### A subset of lncRNAs may be essential

Intersecting our lncRNA sequencing dataset with that of the *piggyBac* transposon mutagenesis study from Zhang et al. determined that 68% (1602) of lncRNAs are predicted to be non-essential in asexual blood stages (Additional File 2: Supp. Fig. 4) [48]. In contrast, no *piggyBac* insertions were found in the remaining 32% (767) lncRNA sequences, suggesting that these lncRNAs may be essential. Among these, 432 are antisense to or overlapping genes deemed essential, meaning we cannot disentangle the essentiality of the protein-coding gene and the lncRNA. The remaining 335 lncRNAs are not associated with essential genes, and thus may have potentially critical functions for parasite growth and viability although the absence of insertions does not definitively demonstrate essentiality (Additional File 3).

### Two novel lncRNAs associated with *var* genes

**Fig. 6:**
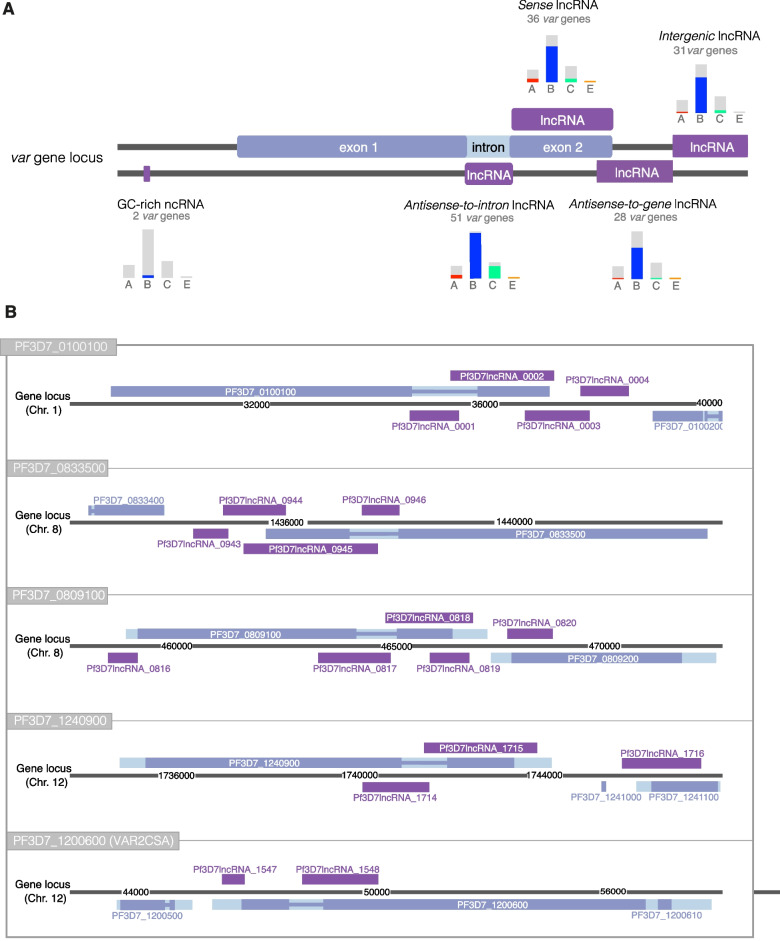
LncRNAs present at *var* gene loci. **a** Schematic of lncRNAs found at *var* gene loci and the number of loci containing each subtype in this study. Bar charts denote the distribution of *var* gene subtypes for these loci by colour (A-red, B-blue, C-green and E-orange). The GC-rich (RUF6) family, *antisense-to-intron* and *sense* lncRNAs are well-established *var*-associated lncRNAs. Two additional lncRNAs; a downstream *intergenic* lncRNA and an *antisense-to-gene* lncRNA were observed in this study. **b** Examples of lncRNAs at five *var* gene loci

Three types of *var*-associated ncRNAs have been described in *P. falciparum*: an *antisense-to-intron* lncRNA, a *sense* lncRNA (overlapping exon 2) and a GC-rich RUF6 ncRNA (usually in a head-to-head configuration and 135 bp in length) (Fig. 6A) [12, 26, 49]. Previous research has suggested that these ncRNAs are widespread in *var* genes but to understand if they are expressed at all *var* loci in mixed asexual blood-stages, we analysed each *var* locus. *Antisense-to-intron* lncRNAs were identified in 51 *var* genes, while *sense* lncRNAs were found in only 36 *var* genes. Only 2 GC-rich RUF6 ncRNAs were detected.

Using CRISPR-interference knockdown, these ncRNAs have been shown to activate the expression of 15 *var* genes *in trans* through predominant transcription of a single member adjacent to the active *var* gene [49, 50]. GC-rich RUF6 ncRNAs PF3D7_0712700 and PF3D7_1240800 were detected in the Lee et al. ONT long-read sequencing dataset, and the latter was adjacent to the single active *var* gene (PF3D7_1240900) [33]. No RUF6 ncRNAs were detected in our sequencing data however, the active *var* gene (PF3D7_1200600, also known as *var2csa*) is not proximal to a RUF6 ncRNA [51]. We also identified two additional lncRNAs at *var* loci that had not been previously described. A downstream *intergenic* lncRNA was detected close to 31 *var* genes and an *antisense-to*-*gene* lncRNA (antisense to exon 2) was detected in 28 *var* genes (Fig. 6A). Examples of lncRNAs at specific *var* gene loci are shown here (Fig. 6B).

## Discussion

Long noncoding RNAs have been shown to be involved in regulating developmental pathways and immune evasion strategies in the malaria parasite *Plasmodium falciparum* [22, 26]. Evidence suggests that there are thousands of genes encoding lncRNAs in the *P. falciparum* genome, but due to limited research and the lack of necessary experimental tools, our understanding of their wider role remains poor [21, 32]. In this study, we sought to provide a basis for future research into these elements by improving their annotation in the *P. falciparum* genome.

We employed manual curation, an approach that has not previously been used for *P. falciparum* lncRNAs, in combination with long-read sequencing to generate a more comprehensive annotation of lncRNAs. Long-read sequencing provided a clear improvement in capturing full-length lncRNAs, by enabling the accurate determination of lncRNA boundaries and providing sequence coverage for lncRNAs that were not captured previously by short-read sequencing. For instance, we identified several lncRNAs that had previously been annotated as multiple lncRNA units. We also expanded the annotation significantly, suggesting that the total number of lncRNAs in *P. falciparum* is over two thousand, which is in line with recent transcriptomic studies that have predicted thousands of potentially noncoding RNA transcripts [31, 32]. Furthermore, our manual curation allowed us to harness the plethora of publicly available datasets to create high-quality genome annotations. Context and supportive evidence were investigated to facilitate each annotation and reduce errors common to automated annotation.

Our characterisation of the genomic and sequence features of lncRNAs largely validates the findings in the field. LncRNAs are widespread throughout the genome, often found at sites with bidirectional promoters and their sequences are AT-rich, vary in length and contain few known RNA motifs. We introduced a genome context-specific classification system, in place of the simplified intergenic and antisense lncRNA system. This allows rich information on the genome context of each lncRNA, which provides a helpful tool for wet lab applications. For instance, in experiments targeting lncRNAs for genetic modification, the off-target effects are a major concern, and presenting contextual subtypes may enable differing approaches to be refined for in vitro study. The subtype classification also allowed for genes associated with certain lncRNA subtypes to be identified using gene ontology. It is evident that *sense* and *antisense-to-intron* lncRNAs are subtypes that are almost exclusive to *var* genes, barring nine additional genes with *antisense-to-intron* lncRNAs, three of which are rifins and one is a *var* pseudogene. The other subtypes are associated with various genes but are enriched for certain biological processes. Interestingly, the *antisense-to-gene* lncRNA subtype was enriched for genes involved in multiple nucleoside and nucleotide processes, with almost all genes labelled with these and related GO terms contextually-associated with a lncRNA of this subtype. Genes involved in protein processes and cell-cell adhesion were also enriched in the *antisense-to-gene* lncRNA subtype, such as the new lncRNAs that we identified at *var* loci. Genes enriched in the *antisense-to-UTR* lncRNA subtype were involved in cytoplasmic translation, immune system processes, chromatin organisation and transport, which included most proteins involved in translation such as the elongation initiation factor (EIF) genes, ribonucleoproteins and epigenetic proteins like histones. LncRNAs could be involved in the regulation of these biological processes and others, and studies on transcriptional expression and biological interactions are required to define these possible roles.

Most of the well-studied lncRNAs from the literature were verified in this study although we did not fully capture the lncRNA-TAREs. These lncRNAs could have been absent due to the stage-specificity of their expression. LncRNA-TARE expression peaks during parasite invasion and therefore, a mixed culture would not be expected to contain large numbers of these parasites [13, 21]. There could also have been challenges in mapping their highly repetitive sequences. The three lncRNA-TAREs that were observed were much shorter in length than expected (Additional File 2: Supp. Fig. 5). However, these short transcripts could be explained by alternative transcription or post-transcriptional processing, which has been observed in lncRNA-TAREs [13, 25]. Sequencing more deeply with long reads and from a wide range of life stages would likely capture these lncRNAs and improve the annotation further.

It has been suggested that some genes, which resemble lncRNAs could encode short polypeptides [32]. We identified a small subset of 16 lncRNAs that have a predicted high coding probability and warrant further investigation. We also identified lncRNAs that could be classified based on containing shorter structural ncRNAs such as snoRNAs, snRNAs and tRNAs. Although these structural ncRNA-lncRNAs hybrids have not been previously reported in *P. falciparum*, they have been observed in other species. In humans, snoRNAs at the Prader-Willi Syndrome locus have been shown to exist as sno-lncRNAs (lncRNA flanked by two snoRNAs) and SPA-lncRNAs (5′ snoRNA capped and 3′ polyadenylated lncRNA), which play a role in post-transcriptional processing of snoRNAs and regulate mRNA metabolism through association with RNA-binding proteins [42, 44], respectively. The lncRNAs identified in this study could play a similar role in the regulation of these structural ncRNAs that are involved in mRNA metabolism and protein synthesis. Or even more simply, these lncRNAs could be processed into snoRNAs in a way similar to genes that contain snoRNAs that splice out and process the snoRNAs from pre-snoRNAs. Further investigation is needed to determine if there is a role for these lncRNAs.

Further work is needed to define the roles that lncRNAs play in the *P. falciparum* transcriptome. Like coding genes, lncRNAs display dynamic regulation across asexual blood stages but little is known about their regulation across other stages of the parasite lifecycle [21]. Studies examining other *P. falciparum* stages such as gametocytes and liver-stages using long-read sequencing are necessary to provide a more complete lncRNA annotation and a better understanding of their regulation and potential functional roles. Extensive in vitro studies are also required to validate the presence of these lncRNAs and subsequently, characterise their features and elucidate their functions. LncRNAs have many possible mechanisms to regulate gene expression and new advances in CRISPR technology may enable the deciphering of the specific functions of *P. falciparum* lncRNAs. This lncRNA annotation will support future studies by providing high-quality sequence annotations that can be used to facilitate functional characterisation such as genome editing, fluorescence labelling, RNA tagging and bioinformatic analyses, leading to an improved understanding of their role in transcriptional regulation.

## Materials and methods

### Parasite culture

*P. falciparum* parasites (3D7 strain) were grown as asexual blood-stage cultures in RPMI media with AlbuMAX® (Gibco) and supplemented with GlutaMax® (Gibco), Gentamicin (Gibco) and HEPES (pH 7) with O^+^ human erythrocytes at 3% haematocrit. Cultures were maintained at 37 °C in a gaseous environment of 3% CO_2_, 1% O_2_ and 96% N_2_. Parasitemia and stages were monitored using Giemsa staining and microscopy. Parasite samples for long-read RNA-seq were harvested from a mixed-staged Pf3D7 culture. Parasite samples for short-read RNA-seq were harvested from synchronised Pf3D7 cultures at different time points around the intra-erythrocytic development cycle (0, 8, 16, 24, 32, 40 and 48 hours) with four replicates for each time point. RNA was extracted from parasites using Trizol as previously described [52].

### Long and short-read RNA sequencing

Short-read libraries were prepared using the Illumina TruSeq kit. They were sequenced on an Illumina HiSeq (ENA project ERP104547) as 150 bp paired-end reads and were mapped to the Pf3D7 reference genome (v3) using HISAT2 v2.0.0 (−-rna-strandness RF, −-max_intronlen 5000) [2, 53]. Two long-read libraries (with and without exonuclease treatment) were prepared by running the Pf3D7 RNA samples on the Oxford Nanopore GridION using the direct RNA-seq protocol, avoiding PCR amplification (ArrayExpress E-MTAB-11766). Exonuclease treatment (TEX) with exonuclease 2 spiked in was used to enrich for primary transcripts as sequencing from both libraries was later combined. Raw data from exonuclease treated and untreated RNA samples in .fast5 files were converted into fastq files of reads using the basecaller Guppy v3.1.5 (−q 0, −r –u_substitution, −-config rna_r9.4.1_70bps_fast.cfg). The exonuclease-treated (TEX plus) sample yielded 55,130 reads. The untreated (TEX minus) sample yielded 377,999 reads. The reads were then mapped against Pf3D7 v3 reference (plus the enolase 2 gene sequence, which is spiked into samples as a control) using minimap2 (−x splice, −G 5000) [37, 54]. The two sets of reads were then merged and used for annotation. The median length of the combined read set was 852 bp, with the longest read being 12,084 bp.

### Data collation, curation, and visualisation

Previous lncRNA annotations were obtained from Liao et al.*,* Broadbent et al. and PlasmoDB [14, 21, 37]. For the Chappell et al. and Yang et al. studies, which predicted lncRNAs but did not generate consolidated annotations, the predicted transcripts were obtained from the supplemental material and the authors, respectively [31, 32]. For the Siegel et al. study, which identified genes with antisense transcription, gene IDs of genes with natural antisense transcripts were derived from the publication due to the absence of antisense transcript coordinate information [18]. Additional RNA sequencing datasets were downloaded from PlasmoDB including long-read ONT RNA-sequencing (Lee et al.), transcriptional start site (TSS) RNA-sequencing (Kensche et al., Chappell et al., Adjalley et al.) and ATAC-seq (Ruiz et al.) [31, 33–36]. The *Plasmodium falciparum* 3D7 reference genome (v3) and annotation (May 2020) were downloaded from the Sanger FTP server (https://www.sanger.ac.uk/resources/downloads/). Sequences were viewed using Artemis, with separate windows created for GC content, long-read and short-read sequencing datasets, TSS datasets, and genome annotations [55].

### Manual annotation of lncRNAs

LncRNAs were manually annotated using the long-read sequence data. LncRNAs were defined as noncoding RNAs of at least 200 nucleotides in length that were not otherwise annotated as another type of noncoding RNA (rRNAs, tRNAs, snRNAs and snoRNAs). One exception was lncRNAs that contained other ncRNAs; however, these transcripts had to be distinctly different from the annotated ncRNA transcripts. The lncRNA boundaries were defined as the outermost positions of the set of reads. LncRNAs were characterised into genomic context subtypes determined by the presence of overlapping (on the same strand), antisense (on the opposing strand) or nearby (within 150 bp) genomic features (Fig. 2). One hundred fifty bp was selected based on previous methods suggesting some UTRs may extend 100 nt or more beyond the position predicted by sequence coverage [31]. LncRNAs were assigned an evidence-based ranking score from 1 to 9 based on three criteria: the presence of the lncRNA in the long-read RNAseq datasets (one or both), number of reads (single vs multiple) and finally, evidence of a distinct TSS in the TSS datasets (none, one or multiple datasets) (Additional File 2: Supp. Fig. 1). TSSs were also used to determine the bidirectionality of promoters. If there was evidence of TSSs on both strands at the same location and expressed at the same time point in the parasite lifecycle then the lncRNA was labelled as potentially driven by a bidirectional promoter.

### Sequence, structure, and coding potential analyses

The comparative analyses with other annotations were completed using Bedtools [56]. Location-based clustering of lncRNAs by subtype was completed using Cluster Locator (v1, max-gap = 2) [57]. Gene ontology (GO) enrichment analyses for antisense and overlapping genes were completed in PlasmoDB (v56) and visualised using REVIGO (v1) [37, 38]. A motif and RNA families search was completed using Rfam (v14.7) batch search [39]. Seqkit was used to obtain AT content information and length, and the presence of exons was determined during annotation [58]. Coding-potential, based on intrinsic sequence features, was analysed using Coding Potential Calculator (v2) and putative proteins were queried in BLAST against all other proteins (blastp and tblastn, v2.12.0) and Pfam (v35.0) and aligned using Clustal Omega [40, 59–61]. Ribosomal footprints were observed in MochiView (v1.46) [62]. Plots were created in R using ggplot2 (v3.3.5), UpSetR (v1.4.0) and idiogramFISH (v1.16.1) packages or using webserver sankeyMATIC [63–65].

## Supplementary Information

**Additional file 1: Supplementary Table 1**. Sequence information. **Supplementary Table 2.** Comparison of some previous annotations to new *P. falciparum* lncRNA annotation. **Supplementary Table 3.** Genomic location-based clustering of *P. falciparum* lncRNAs by subtype. **Supplementary Table 4.** Noncoding motif analysis of *P. falciparum* lncRNAs. **Supplementary Table 5**. Sequence intrinsic features of *P. falciparum* lncRNAs determined to have coding potential.

**Additional file 2: Supplementary Fig. 1**. Evidence ranking based on supportive evidence of lncRNA annotations. **Supplementary Fig. 2**. Examples of lncRNAs that contain multiple introns. **Supplementary Fig. 3**. Putative proteins from lncRNAs with predicted coding potential share sequence similarity with hypothetical proteins from other *P. falciparum* strains. **Supplementary Fig. 4**. The majority of lncRNAs can be disrupted by the *piggyBac* transposon system. **Supplementary Fig. 5**. LncRNA-TAREs were not fully captured by the long-read sequencing.

**Additional file 3: Supplemental File 1**. File containing additional information about lncRNA annotations.

## Data Availability

The data underlying this article are available at ArrayExpress (long-read RNA sequencing, E-MTAB-11766) and European Nucleotide Archive (short-read RNA sequencing, ERP104547). The annotation is available on PlasmoDB.
