# Correction: A manually curated annotation characterises genomic features of *P. falciparum* lncRNAs

**DOI:** 10.1186/s12864-023-09164-0

**Published:** 2023-04-06

**Authors:** Johanna Hoshizaki, Sophie H. Adjalley, Vandana Thathy, Kim Judge, Matthew Berriman, Adam J. Reid, Marcus C. S. Lee

**Affiliations:** 1 Wellcome Sanger Institute, Wellcome Genome Campus, Hinxton, Cambridge, CB10 1SA UK; 2 Micrographia Bio, London, W12 0BZ UK; 3 MRC Weatherall Institute of Molecular Medicine, University of Oxford, Oxford, OX3 9DS UK; 4 Present address: Department of Microbiology and Immunology, Columbia University Medical Center, New York, NY10032 USA; 5 Wellcome Centre for Integrative Parasitology, University of Glasgow, Glasgow, G12 8TA UK; 6 Present address: Wellcome/Cancer Research UK Gurdon Institute, University of Cambridge, Cambridge, CB2 1QN UK

**Correction: BMC Genomics 23, 780 (2022)**

**https://doi.org/10.1186/s12864-022-09017-2**

Following the publication of the original article [1], the authors detected a typo under the heading **Some lncRNAs contain structural RNA sequences.** The correction has been marked in **bold**.

**Some lncRNAs contain structural RNA sequences**

Searches against the RNA families database (Rfam) revealed that 19 lncRNAs contained sequences associated with 22 **unique** described RNA families (Fig. 5A, Additional File 1: Supp. Table 4), including those encoding known structural RNAs such as the signal recognition particle RNA, the ribozyme ribonuclease P and several RNAs of unknown function (RUFs) [41].

Moreover, it was noted that the Figs. 1, 2, 3, 5 and 6 and their legends were not updated. The correct figures are included here. The corrections in the legends were marked in **bold**.

**Fig. 1:**
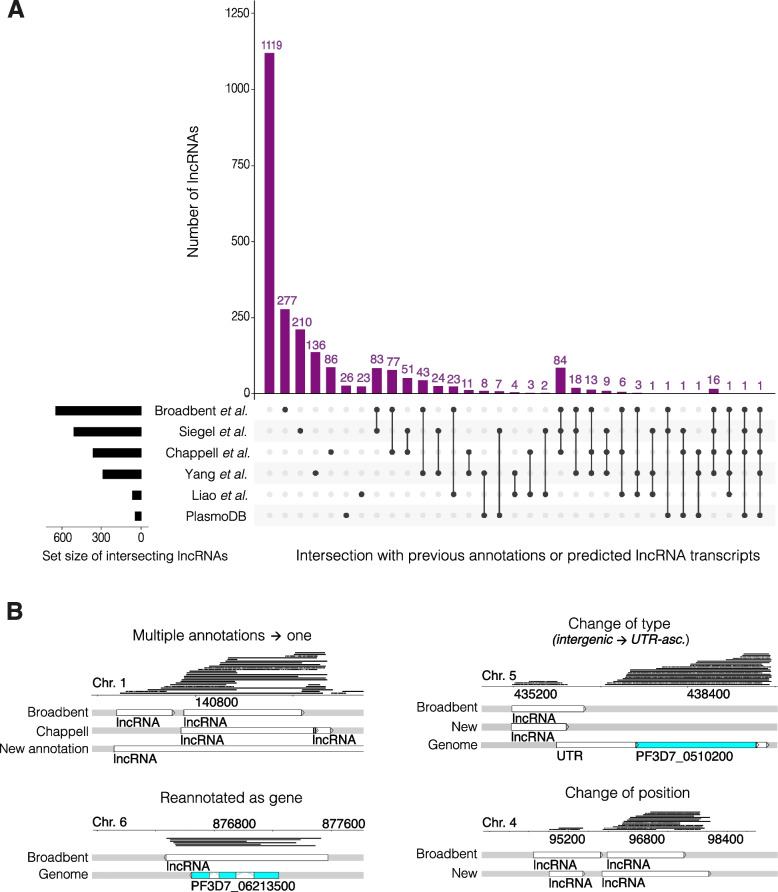
Verifcation of previous P. falciparum lncRNA annotations in the literature. Of the 2369 lncRNAs, 1119 were unique to this study and 1250 were previously annotated by Broadbent et al. or Liao et al., or predicted by Siegel et al., Chappell et al., Yang et al., and/or listed on PlasmoDB from various published studies [14, 18, 21, 31, 32, 37]. **a** An upset plot shows the number and membership of the previously annotated lncRNAs as well as the size of the set. Gene IDs from the Siegel et al. dataset were intersected with gene IDs of genes antisense to lncRNAs in this work [18]. **b** Snapshots demonstrate examples of changes to previous annotations

**Fig. 2:**
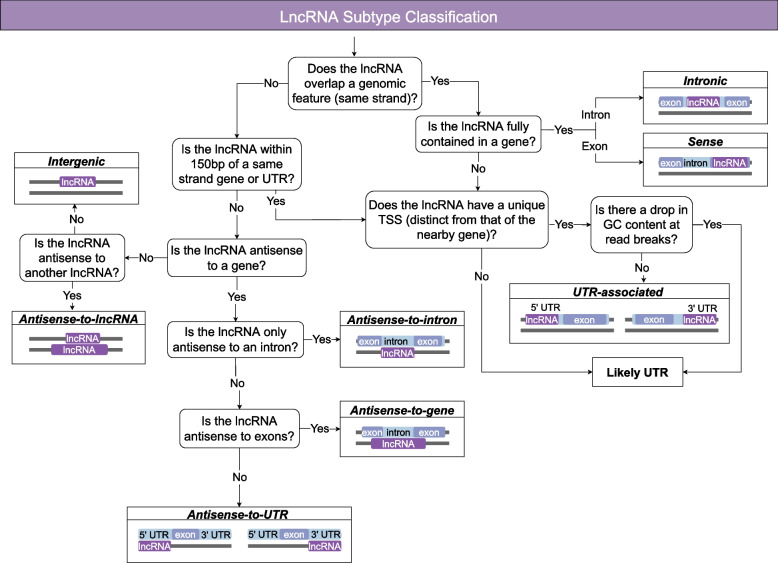
Schematic representation of the classifcation of lncRNA into genome context-based subtypes. Annotations were categorised by genomic context using a decision tree. LncRNAs that overlapped a gene on the same strand were classifed as either intronic if contained within the intron or sense if contained within a single exon. No lncRNAs were annotated that spanned multiple exons in a gene. LncRNAs that overlapped a UTR and lncRNAs nearby genes (within 150bp of an annotated UTR or exon or read from the gene) were fagged as potential UTR-associated lncRNAs. To delineate UTR-associated lncRNAs from UTR transcripts (that could be fragmented due to drops in GC content or alternative start sites) careful examination of collative data was performed. This included an analysis of the level of overlap between reads from the putative lncRNA and gene/UTR, the presence of a unique transcriptional start site (distinct from the gene) and the lack of evidence of a drop in GC content. LncRNAs that were antisense (opposite strand) to genomic features were classifed based on the type of antisense genomic feature: antisense-to-gene, antisense-to-intron, antisense-to-UTR and antisense-to-lncRNA. The antisense-to-intron lncRNAs were contained within the intron boundaries (with little to no overlap with the exon). The antisense-to-UTR lncRNAs only overlapped the UTR, not the exons and the level of overlap varied. Some lncRNAs could be classifed as multiple subtypes if overlapping multiple features – the classifcation has a hierarchy starting with: intronic, sense, UTR-associated, antisense-to-intron, antisense-to-gene, antisense-to-UTR and antisense-to-lncRNA. LncRNAs not overlapping, antisense to, or nearby (150bp) any feature were classifed as intergenic

**Fig. 3:**
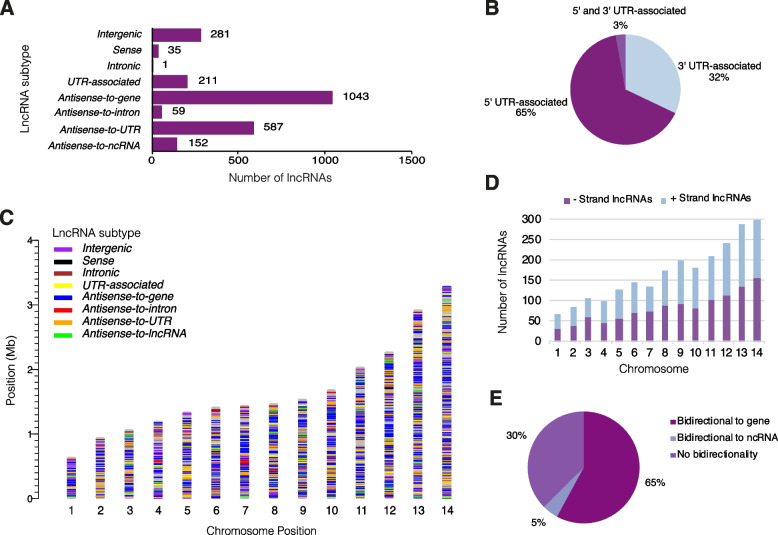
Genomic features of P. falciparum lncRNAs. **a** The majority of lncRNAs were antisense-to-gene lncRNAs or antisense-to-UTR lncRNAs, followed by intergenic lncRNAs, UTR-associated lncRNAs and antisense-to-lncRNA lncRNAs. A minority were antisense-to-intron, intronic or sense lncRNAs. **b** Antisense-to-UTR lncRNAs were most commonly (65%) associated with a 5′ UTR, followed by 32% associated with a 3′ UTR and a small subset (3%) were nestled between 5′ and 3′ UTRs. **c** LncRNAs and their genome-context subtypes were distributed throughout the genome. **d** LncRNAs were equally distributed between positive and negative strands and their abundance in chromosomes was relative to chromosome size. **e** For lncRNAs with an associated transcriptional start site, **70%** had evidence of bidirectionality (transcription in both directions in the same location and time point)

**Fig. 5:**
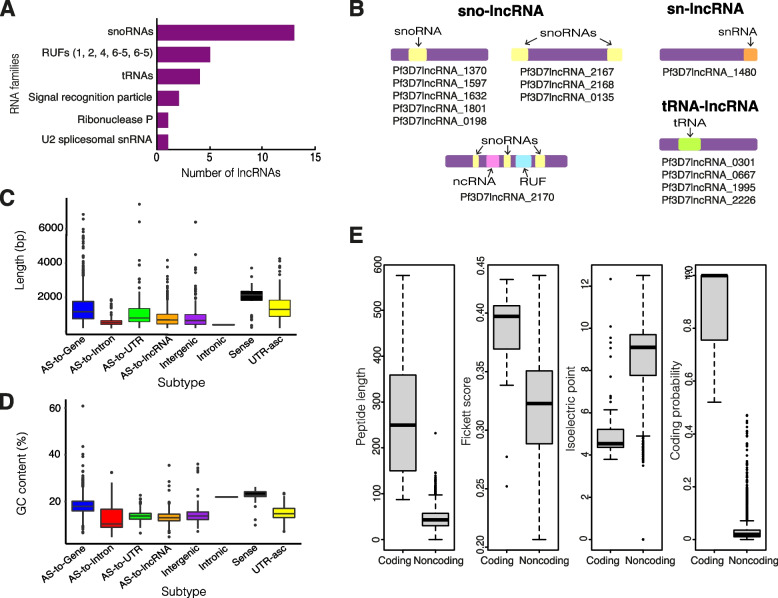
Sequence features of P. falciparum lncRNAs. **a** 26 RNA families (22 unique) were identified that aligned to annotated lncRNA sequences: 13 snoRNAs, 5 RUFs, 4 tRNAs, ribonuclease P, **2 signal recognition peptides** and U6 spliceosomal snRNA using Rfam [39]. **b** Visual representation and IDs of lncRNAs containing snoRNAs, snRNAs and tRNAs. **c** LncRNA lengths ranged from 200 to 7452 bp and the distribution of lengths differed between subtypes (AS:antisense to). **d** LncRNAs were AT-rich with an average GC content of 15.97% and the distribution of GC content differed between subtypes (AS:antisense to). **e** 16 lncRNAs were identified as putatively coding by sequence feature-dependent coding potential analysis with CPC2 [40]. The distributions of the measures used to determine coding potential (peptide length, isoelectric point and Fickett score) are presented for putative coding and noncoding lncRNA annotations

**Fig. 6:**
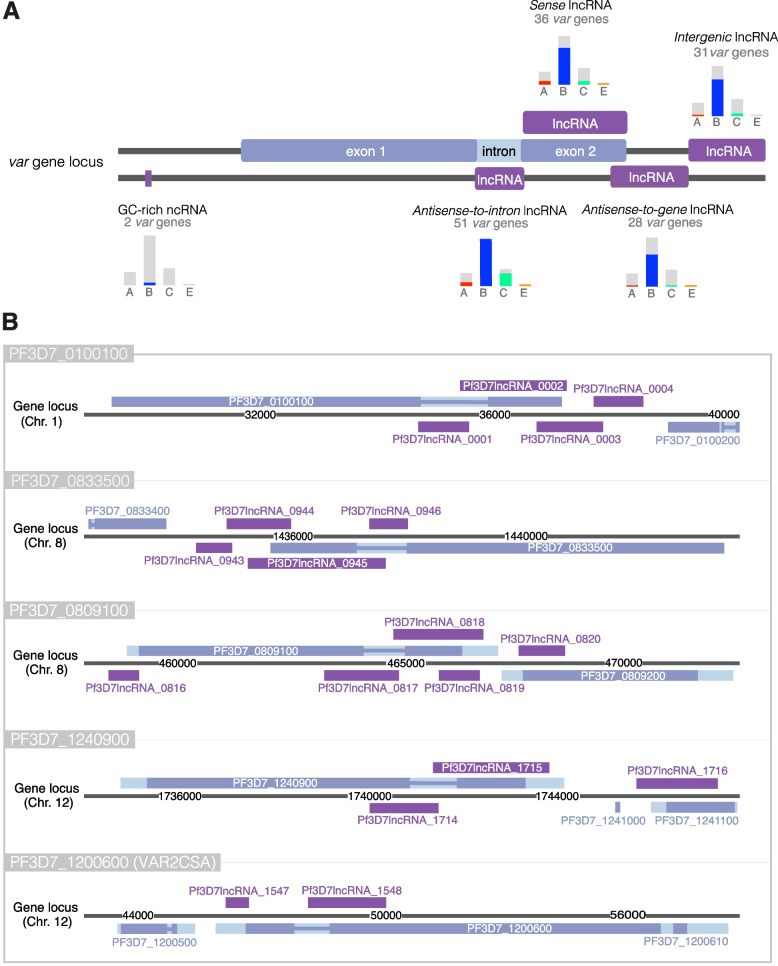
LncRNAs present at var gene loci. **a** Schematic of lncRNAs found at var gene loci and the number of loci containing each subtype in this study. Bar charts denote the distribution of var gene subtypes for these loci by colour (A-red, B-blue, C-green and E-orange). The GC-rich (RUF6) family, antisense-to-intron and sense lncRNAs are well-established var-associated lncRNAs. Two additional lncRNAs; a downstream intergenic lncRNA and an antisense-to-gene lncRNA were observed in this study. **b** Examples of lncRNAs at fve var gene loci

The original article [1] has been updated.
