# Supplementary material for: A manually curated annotation characterises genomic features of P. falciparum lncRNAs
Source: BMC Genomics. 2022 Nov 30;23:780. doi: 10.1186/s12864-022-09017-2 (PMC9710153; doi:10.1186/s12864-022-09017-2)
Supplement: Supplementary file 1 — Additional file 1: Supplementary Table 1. Sequence information. Supplementary Table 2. Comparison of some previous annotations to new P. falciparum lncRNA annotation. Supplementary Table 3. Genomic location-based clustering of P. falciparum lncRNAs by subtype. Supplementary Table 4. Noncoding motif analysis of P. falciparum lncRNAs. Supplementary Table 5. Sequence intrinsic features of P. falciparum lncRNAs determined to have coding potential. [file 12864_2022_9017_MOESM1_ESM.pdf]

Supplemental Table 1. Sequence information

Dataset	N50 read length (bp)	Longest read (bp)	Total sequence (Mbp)
Pf nanopore 1	1306	10245	375.7
Pf nanopore 2*	2008	12804	277.7
Pf short read	2x75 pe	75	24228_11 (1203.7), 24228_25 (1140.7), 24228_13 (1193.5), 24228_20 (1112.2), 24228_14 (1531.2), 24228_18 (1187.2), 24228_16 (1194.1)

*Lee *et al.* dataset [33].

Supplemental Table 2. Comparison of some previous annotations to new *P. falciparum* lncRNA annotation

Comparison to new annotation		Number of previous annotations (by source)		
		Broadbent *et al*. [21]	Chappell *et al.* [31]	PlasmoDB [37]
Change of subtype	*intergenic → UTR-asc.*	64	N/A	N/A
	intergenic → antisense-to-gene	42		
	intergenic → antisense-to-UTR	108		
	antisense-to-gene → UTR-asc.	38		
	antisense-to-gene → intergenic	1		
Change of position	start site expanded	433	327	17
	start site reduced	211	33	7
	stop site expanded	315	310	20
	stop site reduced	332	49	4
Change in number of annotations	single → multiple	1	0	0
	multiple → single	49	543	6
Not classified as a lncRNA	reannotated as gene	3	0	0
	reannotated as UTR	180	39	4
	less than 200bp	15	4	3
Not observed		240	4493*	14

*Represents the number of ncRNA calls in place of annotated lncRNAs.

Supplemental Table 3. Genomic location-based clustering of *P. falciparum* lncRNAs by subtype

LncRNA subtype	Cluster size	Number of clusters	Number of lncRNAs	Percentage of lncRNAs (within subtype)		p-value (compared with random clustering)
				Per cluster (%)	Total (%)	
Intergenic	3	7	21	7.42	34.28	3.09e-07
	4	2	8	2.83		
Antisense-to-gene	3	47	141	13.43	87.90	2.99e-02
	4	23	92	8.76		
	5	2	10	0.95		
Antisense-to-intron	3	1	3	6.13	18.37	3.73e-05
Antisense-to-UTR	3	30	90	15.31	39.80	9.07e-02
	4	3	12	2.04		
	5	2	10	1.70		
Antisense-to-lncRNA	3	5	15	9.93	63.55	< 1.00e-10
	4	1	4	2.65		
	5	1	5	3.31		

Cluster Locator was used to identify positional-based clusters of lncRNAs based on genome position [57]. A max gap, the number of genes in between cluster elements was assigned a value of 2. Clusters of two lncRNAs were excluded. The intronic subtype was not included as it only contains one lncRNA.

Supplemental Table 4. Noncoding motif analysis of *P. falciparum* lncRNAs

Feature	Family ID	Family	Total hits	LncRNAs
Signal recognition particle	RF00017	Metazoa_SRP	1	Pf3D7lncRNA_2143
	RF01856	Protozoa_SRP	1	Pf3D7lncRNA_2143
Ribonuclease P	RF01577	RNase_P	1	Pf3D7lncRNA_0134
RNA of unknown function	RF01578	RUF1	1	Pf3D7lncRNA_1888
	RF01579	RUF2	1	Pf3D7lncRNA_0135
	RF01582	RUF4	1	Pf3D7lncRNA_2170
	RF01581	RUF6-5	2	Pf3D7lncRNA_0063
				Pf3D7lncRNA_0501
U2 snRNA	RF00004	U2	1	Pf3D7lncRNA_1480
snoRNAs	RF01583	snoR01	1	Pf3D7lncRNA_0135
	RF01585	snoR07	1	Pf3D7lncRNA_1370
	RF01590	snoR14	1	Pf3D7lncRNA_1597
	RF01591	snoR15	1	Pf3D7lncRNA_1632
	RF01593	snoR16	1	Pf3D7lncRNA_1801
	RF01598	snoR23	1	Pf3D7lncRNA_2167
	RF01599	snoR24	1	Pf3D7lncRNA_2167
	RF01602	snoR27	1	Pf3D7lncRNA_2168
	RF01604	snoR28	1	Pf3D7lncRNA_2168
	RF01603	snoR29	1	Pf3D7lncRNA_2170
	RF01605	snoR30	1	Pf3D7lncRNA_2170
	RF01606	snoR31	1	Pf3D7lncRNA_2170
	RF00133	SNORD33	1	Pf3D7lncRNA_0198
tRNA	RF00005	tRNA	4	Pf3D7lncRNA_0301
				Pf3D7lncRNA_0667
				Pf3D7lncRNA_1995
				Pf3D7lncRNA_2226

LncRNAs containing known RNA families were identified using Rfam batch search [39].

Supplemental Table 5. Sequence intrinsic features of *P. falciparum* lncRNAs determined to have coding potential

LncRNA	Peptide length	Fickett score	Isoelectric point	ORF integrity	Coding probability	Ribosomal footprints
Pf3D7lncRNA_0624	576	0.28	9.32	1	1.00	No
Pf3D7lncRNA_0937	255	0.35	5.21	1	1.00	Yes
Pf3D7lncRNA_0817	150	0.40	6.14	1	0.93	Yes
Pf3D7lncRNA_0229	174	0.34	6.70	1	0.92	Yes
Pf3D7lncRNA_0892	134	0.40	7.71	1	0.76	Yes
Pf3D7lncRNA_1445	167	0.34	9.06	1	0.74	Yes
Pf3D7lncRNA_1714	116	0.35	4.58	1	0.69	Yes
Pf3D7lncRNA_0100	103	0.40	4.52	1	0.68	Yes
Pf3D7lncRNA_1391	95	0.39	3.79	1	0.66	Yes
Pf3D7lncRNA_2030	143	0.37	10.08	1	0.62	Yes
Pf3D7lncRNA_0682	87	0.25	3.91	1	0.58	Yes
Pf3D7lncRNA_0260	140	0.35	8.66	1	0.55	Yes
Pf3D7lncRNA_0275	155	0.39	12.33	1	0.55	Yes
Pf3D7lncRNA_1822	121	0.37	6.79	1	0.54	Yes
Pf3D7lncRNA_1890	132	0.37	9.55	1	0.53	Yes
Pf3D7lncRNA_0262	119	0.37	6.51	1	0.52	Yes

LncRNAs with coding potential were identified using Coding Potential Calculator (CPC2) [40]. The features used to make the prediction: peptide length, Fickett score, isoelectric point, ORF length and coding probability, are listed here for the 16 lncRNA determined to have coding potential. Ribosomal footprints from Caro *et al*. were viewed in PlasmoDB and MochiView [37, 47, 62]. A result of “yes” was reported if a mark was present for any timepoint otherwise a result of “no” was reported.
