# Supplementary material for: A manually curated annotation characterises genomic features of P. falciparum lncRNAs
Source: BMC Genomics. 2022 Nov 30;23:780. doi: 10.1186/s12864-022-09017-2 (PMC9710153; doi:10.1186/s12864-022-09017-2)
Supplement: Supplementary file 2 — Additional file 2: Supplementary Fig. 1. Evidence ranking based on supportive evidence of lncRNA annotations. Supplementary Fig. 2. Examples of lncRNAs that contain multiple introns. Supplementary Fig. 3. Putative proteins from lncRNAs with predicted coding potential share sequence similarity with hypothetical proteins from other P. falciparum strains. Supplementary Fig. 4. The majority of lncRNAs can be disrupted by the piggyBac transposon system. Supplementary Fig. 5. LncRNA-TAREs were not fully captured by the long-read sequencing. [file 12864_2022_9017_MOESM2_ESM.pdf]

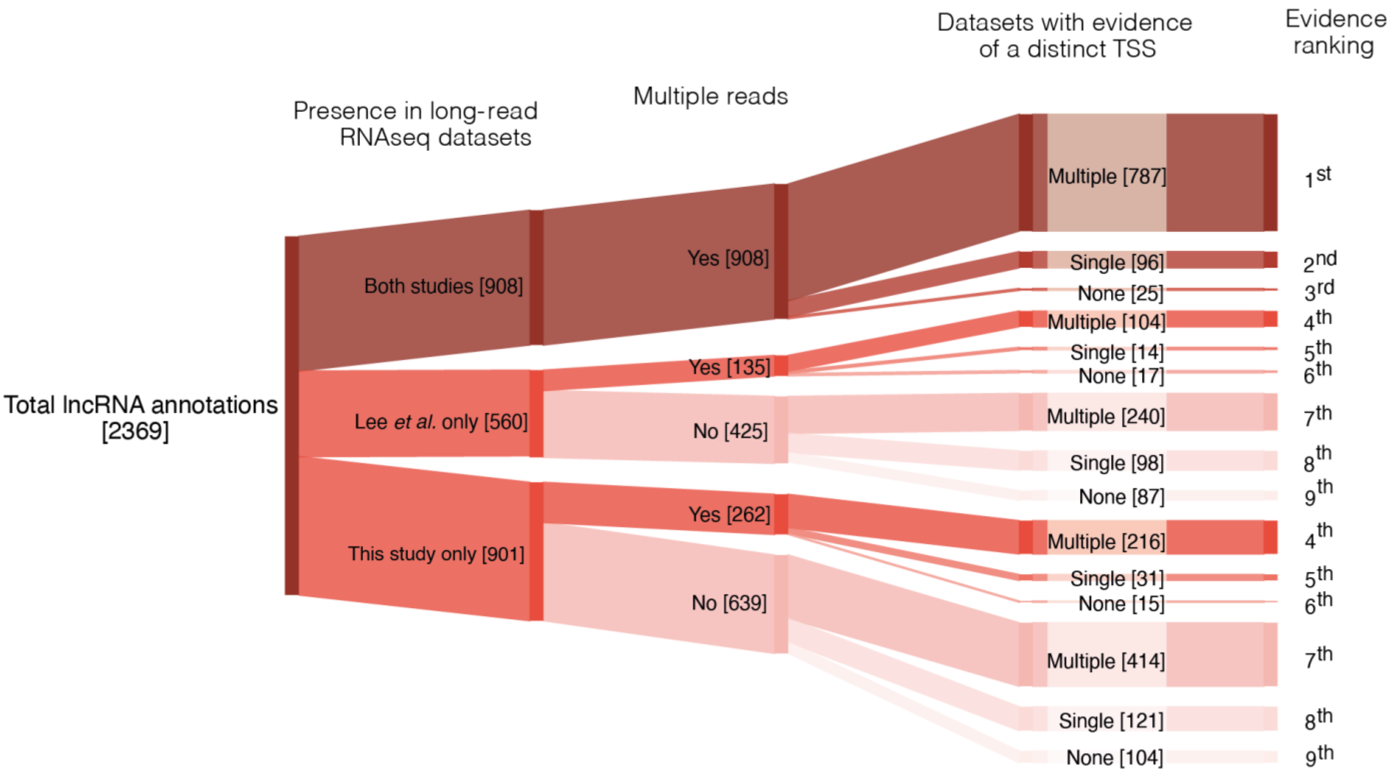

Supplementary Figure 1. Evidence ranking based on supportive evidence of lncRNA annotations. LncRNAs were assigned a ranking from 1-9, 1st representing lncRNAs with the most evidence and 9th representing lncRNAs with the least evidence. Three criteria were used to define the ranking: 1- Presence in both vs one of the long-read RNAseq datasets (our sequencing and Lee *et al.*) [33]; 2- the presence of multiple reads vs a single read; 3- evidence of a distinct TSS from multiple datasets vs a single dataset vs no evidence of a TSS (Adjalley *et al*., Kensche *et al*. and Chappell *et al.* datasets) [31, 34-35]. A Sankey plot shows how these criteria were used to determine rank.

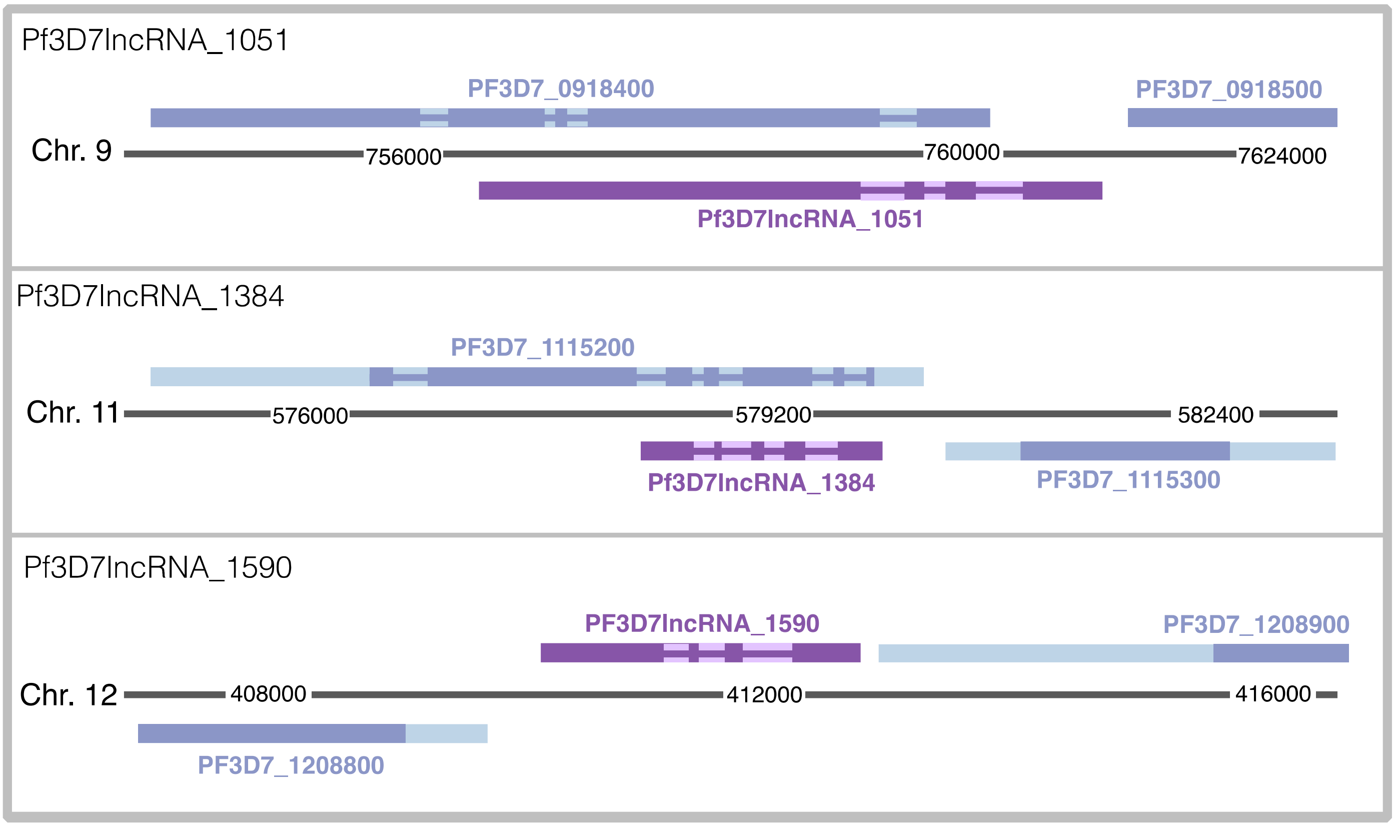

Supplementary Figure 2. Examples of lncRNAs that contain multiple introns. Schematic of the loci of three lncRNAs that contain multiple introns: Pf3D7lncRNA_1051, Pf3D7lncRNA_1384 and Pf3D7lncRNA_1590.

A

```
Pf3D7lncRNA_1391          ------MVIYNNMVIYNNEVIYNNMVIYNNMVIYNNMVIYNNMVIYYNEVIYNNMVIYNNMVIYN------NMVIYNNMVIYNNMVIYYNILIYYYFVNQFFTTSI    94
PFLG_00935 (PF DD2)       ------MVIYNNMVIYNNEVIYNNMVIYNNMVIYNNMVIYNNMVIYYNEVIYNNMVIYNNMVIYNNMVIYNNMVIYNNMVIYYNILIYYYFVNQFFTTSI    100
PFDG_04709 (PF DD2)       MVIYNNMVIYNNMVIYNNEVIYNNMVIYNNEVIYNNMVIYNNMVIYYNEVIYN------------NMVIYNNMVIYNNMVIYNNMVIYYNILIYYYFVNQFFTTSI    94
                                ************************ **********************                  ***********************************
```

B

```
Pf3D7lncRNA_0624      MKVSSQYPVTLRIVESRLEVSSQYPVTLRIVEISREDISQYPGNLRIVERRLEVSSQYPGNLRIVERRLEVSSQYPGNLRIVESSRKDSSQYPGNLRIVEISRKDSSQYPVTLRIVESSR  120
PFDG_04022 (PF Dd2)   --------------------------------------------------------------------------------------------------------------------MVEGSL  6
PFDG_01724 (PF Dd2)   -----------------------SGEKYTVSGNSTDCRDQSGGQFTVPSNSTDCREQAGGGFTVPSNSTDCRDQSGGHFTVPRKS-------------TDCREEAGGQFTVPRKSADCRE  84
KERATIN (PF RAJ116)   MD--------VCIFS------DNTDRIVKVMETNVHGIDQSGGQFTVPSNSTDCRDQFGGQFTVPSNSTDCRDQSGGQFTVPSNS-------------TDCREQAGGQFTVPRKSADCRE  93
THR-SER (PF IGH-CR14) ------------------------------------------------------------------------------------------------------------------------  0

Pf3D7lncRNA_0624      KDSSQYPVTLRIVEICRKDSSQYPVTLRIVEISREDISQYPGNLRIVERRLEVSSQYPGNLRIVERRLEVSSQYPGNLRIVESSRKDSSQYPGNLRIVEISRKDSSQYPGNLRIVE--IS  238
PFDG_04022 (PF Dd2)   EKSTQYLVAVRVVECSLEKSTQYLVAVRVVECSLEKSTQYLVAVRVVECSLEAGGQFTVPSNSTDCRDLSRGQYTVPRKSTDCRDLSRGQYTVPSNSTDCRDQSGGQYTVPSNSTDCRDQ  126
PFDG_01724 (PF Dd2)   QSEGQFTVPRKSTDCREQSEGQFTVPRKSTDCRDQSEGQFTVPSNSTDCRDLSEGQFTVPSNSTDCRDLSEGQFTVPRKSADCRDLSEGQFTVPRKSADCREEAGGQFTVPRKSADCREQ  204
KERATIN (PF RAJ116)   EAGGQFTVPSNSTDCRDQSGGHFTVPRKSADCREEAGGQFTVPRKSADCREEAGGQFTVPRKSTDCREEAGGQFTVPRKSADCRDLSEGQFTVPRKSTDCRDLSEGQFTVPRKSTDCREQ  213
THR-SER (PF IGH-CR14) ----------------------------------------------------------------------MVELPSNSTDCRDQSEGQFTVPSNSTDCRDQSEGQFTVPSNSTDCRDQ  48
                                                                                                    . .:.   . .*:    . .:      .*:    . .:   .

Pf3D7lncRNA_0624      RKDSSQYPVNSTDCREQSEGQFTVPSNSTDCRDLSEGQFTVPSNSTDCRDLSEGQFTVPRKSTDCRDLSRGQYTVPRKSTDCRDQSEGQFTVPRKSTDCREQSGGQFTVPRKSTDCREQS  358
PFDG_04022 (PF Dd2)   SGGQFTVPRKSTDCRDQSEGQFTVPRKSTDCRDQSEGQFTVPRKSTDCRDQSEGQYTVPRKSSDCRDLSRGQYTVPSNSTDCRDLSRGQYTVPSNSTDCREQSGGQFTVPSNSTDCREQS  246
PFDG_01724 (PF Dd2)   SEGQFTVPRKSTDCRDQSEGQFTVPRKSTDCREQSGGQFTVPRKSTDCRDLSRGQYTVPRKSTDCRDLSRGQYTVPRKSTDCRDLSRGQYTVPRKSTDCRDLSRGQYTVPRKSTDYRDQS  324
KERATIN (PF RAJ116)   SEGQFTVPRKSTDCREQSEGQFTVPRKSTDCRDLSEGQFTVPRKSADCRDQSEGQFTV-----------------PRKSTDCRDLSRGQYTVPRKSTDCRDLSRGQYTVPRKSTDCREQS  316
THR-SER (PF IGH-CR14) SEGQFTVPSNSTDCRDQSEGQYTVPRKSTDCRDLSRGQYTVPRKSTDCRDLSRGQYTVPRKSTDCRDLSRGQYTVPRKSTDCREQSGGQYTVPSNSTDCREQSGGQFTVPSNSTDCREQS  168
                        ..   * :*****:*****:*** :*****: * **:*** :*:**** *.**:**                 * :*****: * **:*** :*****: * **:*** :*** *:**

Pf3D7lncRNA_0624      GGQFTVPRKSTDCRDLSRGQYTVPRKSTDCRDLSRGQYTVPR-------KSTDCRDLSRGQYTVPRKSTDCRDLSRGQYTVPRKSTDCRDLSRGQYTVPRKSTDCRDLSRGQYTVPRKST  471
PFDG_04022 (PF Dd2)   GGQFTVPSNSTDCREQSGGQFTVPSNSTDCREQSGGQFTVPS-------NSTDCRDLSRGQYTVPRKSTDCRDLSRGQYTVPSNSTDCREQSGGQFTVPSNSTDCREQSGGQFTVPSNST  359
PFDG_01724 (PF Dd2)   GGHFTVPRKSADCREEAGGQFTVPRKSTDCRDLSRGQYTVPR-------KSTDCRDLSRGQYTVPRKSTDCRDLSRGQYTVPRKSTDCRDLSRGQYTVPRKSTDCRDLSRGQYTIPRKST  437
KERATIN (PF RAJ116)   G-----------------GQFTVPRKSTDCREQSEGQFTVPR-------KSTDCREQSGGQFTVPRKSTDCRDLSRGQYTVPRKSTDCRDLSRGQYTVPRKSTDCRDQLGGQFTVPRKST  412
THR-SER (PF IGH-CR14) GGQFTVPSNSTDCREQSGGQFTVPSNSTDCREQSGGQFTVPRNSTSVPRKSTDCREQSGGQFTVPRKSTDCRDQSEGQFTVPRKSTDCRDQSEGQYTVPRKSTDCRDLSRGQYTVPRKST  288
                      *                 **:*** :*****: * **:***        :*****: * **:*********** *.**:*** :*****: * **:*** :*****:   **:*:* :**

Pf3D7lncRNA_0624      DCRDLSRGQYTVPRKSTDCRDLSRGQYTVPRKSTDCRDQLGGQFTVPSNSTDCREQSGGQFTVPSNSTDCREEAGGQFTVPR-------KSTDCRDQSEGQFTVPSKLYGL---------  575
PFDG_04022 (PF Dd2)   DCRDLSRGQYTVPRKSTDCRDLSRGQYTVPRKSTDCREQSGGQFTVPSNSTDCREQSGGQFTVPRKSTDCREEAGGQFTVPS-------NSTDCRDQSGGQFTVPRNSTSGR--------  464
PFDG_01724 (PF Dd2)   DCRDQSGGQFTVPRKSADCRDLSRGQYTVPRKSTDCREEAGGQFTVPRKSTDYRDQSGGHFTVPRKSTDCRDQSEGQFTVPR-------KSTDCREQSEGQYTVPRKSTDCRDLSRGQYT  550
KERATIN (PF RAJ116)   DCRDQLGGQFTVPRKSTDCRDLSRGQYTVPRKSTDCRDQLGGQFTVPSNSTDCREQSGGQFTVPRKSTDCRDLSRGQFTVPR-------NSTSGRKVWRTVHST-----------QKLYG  514
THR-SER (PF IGH-CR14) DCRDLSRGQYTVPRKSTDCRDLSRGQYTVPSNSTDCREQSGGQYTVPSNSTDCREQSGGQFTVPSNSTDCREQSGGQFTVPRNSTSVPRKSTDCREQSGGQFTVPRKSTDCRDLSRGQYT  408
                      ****   **:******:************* :*****:: ***:*** :*** *:****:**** :*****: : ******        :**. *.     .:.
```

Supplementary Figure 3. Putative proteins from lncRNAs with predicted coding potential share sequence similarity with hypothetical proteins from other *P. falciparum* strains. Sequences were obtained from an NCBI BLASTp search and were aligned using Clustal Omega (ClustalW with character counts) [59-61]. Pf3D7lncRNA_1391 shared sequence similarity with hypothetical proteins PFLG_00935 from *P. falciparum* RAJ116 (e-value: 8e-35) and PFDG_04709 from *P. falciparum* Dd2 (e-value: 3e-28). Pf3D7lncRNA_0624 shared sequence similarity with many hypothetical proteins, a few of which are shown here: PFDG_04022 and PF3D7_01724 from *P. falciparum* Dd2 (e-value: 0), Keratin KB40 from *P. falciparum* RAJ116 (e-value: 0) and Thr-Ser protein *P. falciparum* IGH-CR14 (e-value: 0). The NCBI IDs for these sequences are KNC35879.1, KOB89161.1, KOB87748.1, KOB86616.1, KNC36782.1 and KNG78363.1.

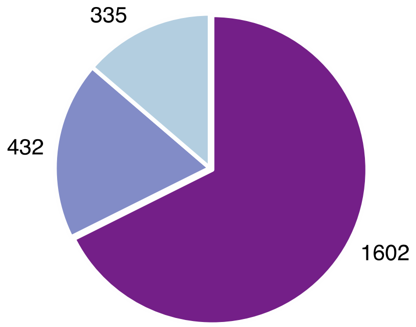

Supplementary Figure 4. The majority of lncRNAs can be disrupted by the *piggyBac* transposon system. The lncRNA annotations were intersected with insertion site positions from surviving mutants in the Zhang *et al.* *piggyBac* transposon mutagenesis screen [48]. 1602 lncRNAs overlapped with insertion positions suggesting that these lncRNAs are not essential, while the remaining 767 lncRNAs did not have any insertions. Of these 767 lncRNAs, 432 are antisense to or overlapping with genes identified as essential in the Zhang *et al.* study and therefore cannot be discerned for essentiality. The remaining 335 are potentially essential lncRNAs.

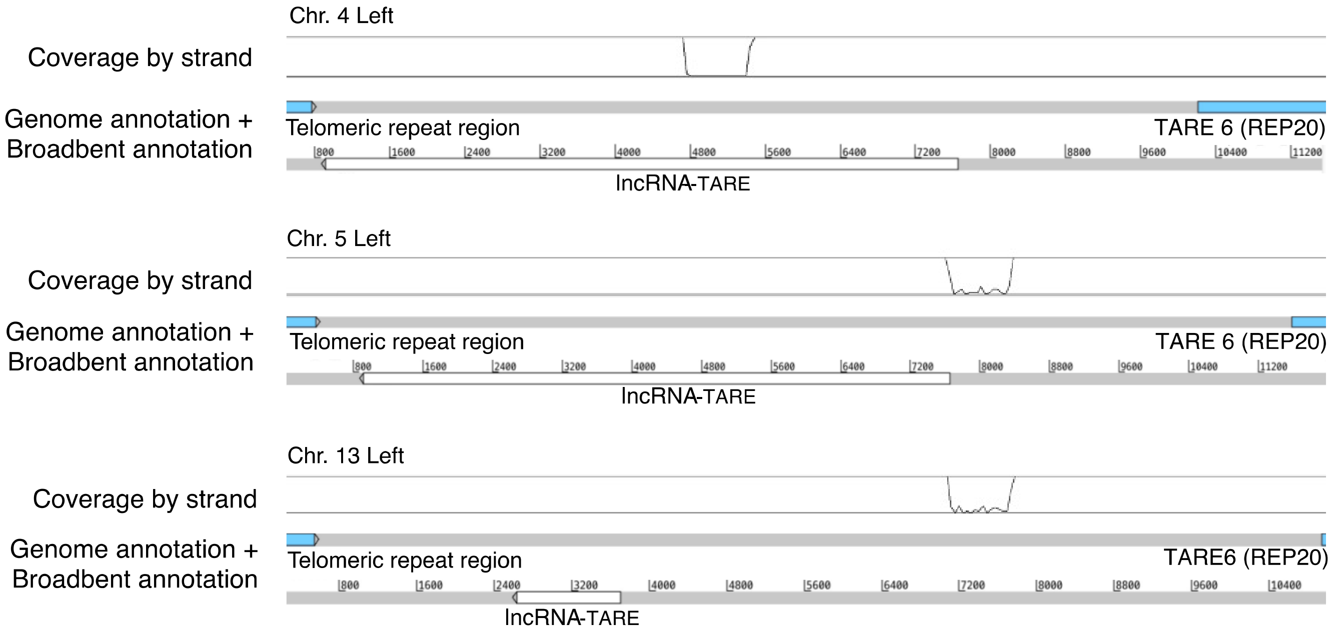

Supplementary Figure 5. LncRNA-TAREs were not fully captured by the long-read sequencing. Three lncRNAs were found in the sub-telomeric region (antisense to the TAREs), however, their length and location differed from the Broadbent annotation, snapshots from Artemis show these lncRNAs [21,55].
